# Physicochemical and Structural Properties of Pomelo Peel Pectin From Majiayou Pomelo With Different Storage Periods

**DOI:** 10.1002/fsn3.70227

**Published:** 2025-06-05

**Authors:** Jian Zhou, Shuoru Feng, Yue Zhou, Zhenghua Huang, Bin Li, Mengting Pi, Siyi Wei, Leipeng Cao

**Affiliations:** ^1^ Academician Workstation, School of Pharmacy Jiangxi University of Traditional Chinese Medicine Nanchang China; ^2^ Department of Pharmacy, First Affiliated Hospital, Jiangxi Medical College Nanchang University Nanchang China; ^3^ Research Institute of Quality, Safety and Standards of Agricultural Product Jiangxi Academy of Agricultural Sciences Nanchang China

**Keywords:** antioxidant activity, majiayou pomelo, metal adsorption, pectin, storage period

## Abstract

Majiayou pomelo peel (MPP) pectin obtained from Majiayou pomelo with different storage periods (0–150 days) was compared based on physicochemical, structural, functional properties and their correlations. The yield, degree of esterification (DE), molecular weight (Mw), particle size, galacturonic acid (GalA), thermal stability, antioxidant activity (AOA), apparent viscosity, and viscoelasticity of MPP gradually decreased with the extension of pomelo storage periods, possibly due to the fracture of the main chain in the pectin molecule. The DE value of MPP gradually decreased to the range of low‐methoxyl pectin (LMP, < 50%) after 150 days of storage probably because of the loss of the –CH_3_ group in the main chain of MPP. The color difference, absolute zeta potential, and metal adsorption capacity of pectin significantly increased with the extension of pomelo storage periods (*p* < 0.05), attributing to the de‐esterification of pectin and decrease of GalA via endo‐polygalacturonase (endo‐PG) activity. Correlation analysis confirmed that high DE, GalA, and low branched Rhamnogalacturonan‐I (RG‐I) region in pectin could benefit from increasing its antioxidant, thermal stability, and rheological properties. These results suggested that the fresh MPP_0_ showed better physicochemical and functional properties for application in pharmaceuticals and cosmetics, and no‐freshness MPP (MPP_60_ and MPP_150_) has good prospects for implementation in foods.

## Graphical abstract

Fresh MPP0 showed better physicochemical and functional properties for application in pharmaceuticals and cosmetics; no‐freshness MPP (MPP60 and MPP150) has good prospects for implementation in foods.

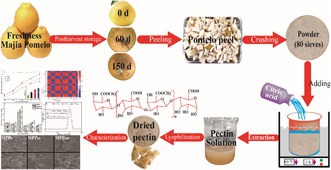

## 1 Introduction

Pectin is an anionic bioactive hetero‐polysaccharide composed of smooth homo‐ and hairy rhamnose‐galacturonic acid glycan domains (Fihry et al. 2022; Yang et al. 2023). The main building unit components of pectin are residues of D‐galacturonic acid (GalA) correlated to α‐1,4 position and several neutral sugars (arabinose, rhamnose, and galactose). Currently, pectin is widely applied in food, pharmaceutical, and cosmetic industries as a gelling agent, antioxidant, and thickener because of the virtues of safety and biodegradability (Zioga et al. 2022; Mada et al. 2022). The degree of esterification (DE) in pectin is named as the proportion of esterified galacturonic acid units to total galacturonic acid units, which is closely associated with its structure and biofunctional properties (Picot‐Allain et al. 2022; Lin et al. 2024). Depending on DE, pectin can be divided into high‐methoxyl pectin (HMP, DE > 50%) and low‐methoxyl pectin (LMP, DE < 50%). Liang et al. (2022) found that alkali de‐esterification could attenuate the gel and emulsifying ability of pectin. Fan et al. (2020) showed that LMP presented better protective effects in acute colitis mice compared to HMP. Shen et al. (2022) demonstrated that hydrogels of LMP had great conduciveness to adsorb copper ions because of finer pores and higher –COOH groups. Currently, many studies focused on the effect of extraction method, raw material, and harvest time on the physicochemical and biofunctional properties of pectin (Fihry et al. 2022; Xiao et al. 2021). However, the influence of raw material storage periods on the physicochemical and functional properties of pectin is unclear.

Majiayou pomelo (*Citrus grandis L. Osbeck*) is a unique and abundant red pulp pomelo in Guangfeng county, Jiangxi, China, which has attracted increasing interest due to its large size, high nutritional value, active substances, unique fragrance, and storability (Jiang et al. 2024; Nie et al. 2019). Majiayou pomelo peel (MP), a directly inedible by‐product constituting 30%–50% (w/w) pomelo, is extensively applied as a raw material source for the extraction of functional compounds (pectin, dietary fiber, essential oils, limonoids, and flavonoids) (Xiao et al. 2021; Lin et al. 2024). The content of pectin in pomelo peel (27.63%) was higher than that of orange peel, apple peel, and banana peels (Chan et al. 2017). Currently, most reports have been dedicated to exploring novel extraction techniques and practical applications of pectin derived from citrus fruits and banana peels, but systematic investigation into the functional and physicochemical characteristics of Majiayou pomelo peel pectin (MPP) remains notably underexplored.

Therefore, this study aims to characterize the physicochemical and functional properties of Majiayou pomelo (MP) peel pectin from mature pomelo, investigate the influences of Majiayou pomelo storage periods on the physicochemical and functional properties of MPP, and explore the correlation between the structure and biofunctional properties of MPP. These results will offer a theoretical guidance for the evaluation of raw materials and standardized production of MPP and realize the highvalue utilization of MP and sustainable development of Majiayou pomelo.

## 2 Materials and Methods

### 2.1 Majiayou Pomelo and Reagents

Thirty mature Majiayou pomelo ( *Citrus grandis* L. Osbeck) (1.5–1.8 kg) with pale‐yellow and no damage were provided by a local orchard in Guangfeng, Jiangxi, China. To minimize postharvest water loss, all pomelo were individually wrapped in polyethylene film immediately after harvesting.

Ascorbic acid, Bovine serum albumin, Coomassie brilliant blue G250, and standard monosaccharides (D‐galacturonic acid [Gal A], D‐glucuronic acid [GlcA], L‐rhamnose, Dglucose [Glc], D‐xylose [Xyl], L‐arabinose (Ara), D‐galactose [Gal], D‐mannose [Man], and L‐Fucose [Fuc]) were obtained from Sigma Chemical Co. Ltd. (USA). ABTS (2,2′‐azino‐bis(3‐ethylbenzothiazoline‐6‐sulfonic acid)) and DPPH (2,2‐diphenyl‐1‐picrylhydrazyl) reagent were obtained from Merck chemical Co. Ltd. (Germany). Other chemical reagents were of analytical grade. All solutions were prepared by using ultrapure water.

### 2.2 Experimental Pre‐Treatment

Thirty freshly harvested Majiayou pomelos (1.5 kg each) were initially wrapped with plastic cling film for preservation and divided into three experimental groups. These groups were subjected to different storage durations (0, 60, and 150 days) at 25°C. Following the completion of the respective storage periods, the pomelos were thoroughly washed and manually peeled. The collected peels were then dehydrated in a forced‐air oven maintained at 55°C until constant weight was achieved. The dried peel samples were subsequently pulverized using an electric grinder and passed through an 80‐mesh standard sieve to ensure particle size uniformity. The resulting powder was aliquoted and stored in airtight containers at 4°C for subsequent analytical procedures.

### 2.3 Pectin Extraction

Pectin was extracted from Majiayou pomelo (MP) peel according to the method of Liu et al. (2023) with a minor modification. The MP powder (60 g) was mixed with citric acid solution (pH 3.0) at a ratio of 1:20 (w/v), and then the mixture was reacted at 90°C for 2 h under continuous stirring. After the reaction, the mixture underwent centrifugation at 3773 *g* for 20 min; the collected supernatant was then twice precipitated with 95% ethanol (1:4, v/v) at 4°C (12 h each). The precipitate was collected via centrifugation at 3773 *g* for 20 min. Finally, the precipitated pectin was lyophilized and milled (80‐mesh), and the pectin powder was named MPP_0_, MPP_60_, and MPP_150_ at 0, 60, and 150 days storage, respectively. The yield of pectin could be evaluated using Equation (1)

(1) $$\text{Yield of pectin}\;\left(\%\right)=W_{0}\times 100/W$$

where *W*_0_ and *W* are the weights of dried pectin (g) and initial dried sample (g), respectively.

### 2.4 Physicochemical Properties of Pectin

#### 2.4.1 Galacturonic Acid (GalA)

Galacturonic acid (GalA) content was quantified using a modified carbazole colorimetric method (Li et al. 2022). Briefly, 1.0 mL of pectin solution (0.1 mg/mL) was thoroughly mixed with 6 mL concentrated H_2_SO_4_ under ice‐cooled conditions, followed by thermal reaction at 85°C for 20 min with continuous shaking. After cooling, 0.2 mL of 1% (w/v) carbazole‐ethanol solution was added, and the mixture was incubated at 25°C for 2 h to complete chromogenic development. Absorbance measurements were performed at 530 nm using a UV‐9000S spectrophotometer (Metash, China). A standard calibration curve was established with GalA solutions (0–100 μg/mL) following identical procedures, using deionized water as the blank reference.

#### 2.4.2 Degree of Esterification (DE)

The degree of esterification (DE) of pectin was determined through an optimized titration protocol (Liang et al. 2022). Briefly, 50 mg of pectin was dissolved in 100 mL distilled water at 50°C, followed by pH adjustment to 8.5 ± 0.2 with 0.1 mol/L NaOH (V_1_) using phenolphthalein indicator. The solution underwent saponification with 10 mL 0.5 mol/L NaOH under vigorous stirring at 30°C for 30 min. Subsequent neutralization was achieved by adding 10 mL 0.5 mol/L HCl until solution decolorization. The residual acidity was then quantified through back‐titration with 0.1 mol/L NaOH (V_2_) to a persistent pale pink endpoint. The DE (%) of pectin was evaluated as follows Equation (2)

(2) $$\mathrm{DE}\;\left(\%\right)=V_{2}\times 100/\left(V_{1}+V_{2}\right)$$

#### 2.4.3 Molecular Weight (Mw) and Distribution

The Mw of pectin was determined using a high‐performance liquid chromatography (HPLC) with a refractive index detector and an xBridge amide column (4.6 × 250 mm, 5 μm, Waters, USA) according to the method of Yu et al. (2021). The pectin and standards (10 mg/mL) were dissolved in 0.1 mol/L NaNO_3_ (mobile phase) and then filtered via a 0.45 μm hydrophilic membrane (PES, Millipore). Filtrates (20 μL) were eluted with 0.1 mol/L NaNO_3_ at a flow velocity of 0.5 mL/min at 35°C for 60 min. The standard curve was created using dextran with Mw of 21, 123.5, 226.7, 420, and 610.5 kDa, respectively. The polydispersity index (PDI) is a key parameter indicating the uniformity of molecular weight distribution, which could be calculated using Equation (3)

(3) $$\mathrm{PDI}=M_{w}/M_{n}$$

where *M*_*w*_ and *M*_*n*_ represent weight average molecular weight and number average molecular weight .

#### 2.4.4 Monosaccharide Composition

The monosaccharide composition of pectin was analyzed by high‐performance liquid chromatography (HPLC) coupled with an evaporative light‐scattering detector (ELSD), employing an XBridge Amide column (4.6 × 250 mm, 5 μm; Waters, USA) according to the method of Fernández‐Delgado et al. (2023). Briefly, 5.0 mg of pectin was hydrolyzed with 4 M trifluoroacetic acid (TFA, 5.0 mL) at 121°C for 1 h. The hydrolysate was subsequently treated with methanol (5.0 mL) and evaporated to dryness under a nitrogen stream. The resulting residue was reconstituted in 5.0 mL ultrapure water and filtered through a 0.45 μm nylon membrane prior to analysis.

Chromatographic separation was achieved under isocratic elution conditions using a mobile phase comprising 85% 0.05 M ammonium acetate (pH 5.2) and 15% acetonitrile (v/v) at a flow rate of 1.0 mL/min. The column temperature was maintained at 50°C, and 20 μL aliquots were injected for each analysis.

The homogalacturonan (HG) and rhamnogalacturonan xI (RG‐I) content were measured as HG (mol%) = GalA (mol%) − Rha (mol%), and RG‐I (mol %) = 2 × Rha (mol %) + Ara (mol %) + Gal (mol%) according to the method of Cybulska et al. (2022).

#### 2.4.5 Color Measurement

The color of pectin was measured by color difference instrument (TS7700, Sanenshi Technology Co. Ltd., Shanghai, China). The color values of L*, a*, and b* were obtained in the CIE LAB system. The changes in color (*∆E*) were evaluated using Equation (4)

(4) $$\Delta E=\sqrt{\left({\left(L*-L_{0}^{*}\right)}^{2}+{\left(a*-a_{0}^{*}\right)}^{2}+{\left(b*-b_{0}^{*}\right)}^{2}\right)}$$

where L* (0–100), a* (from −120 to +120), and b* (from −120 to +120) represent lightness, redness, and yellowness, respectively. $L_{0}^{*}$, $a_{0}^{*}$, and $b_{0}^{*}$ correspond to the color of control groups, respectively.

#### 2.4.6 Particle Size and Zeta Potential

The particle size distribution and zeta potential of pectin solution (1.0 mg/mL) were analyzed using a Zetasizer Nano‐ZS90 instrument (Malvern Instruments, UK) at 25°C, following the established protocol of Li et al. (2023). Measurement parameters were optimized with a pectin refractive index of 1.47 and ultrapure water (refractive index = 1.33) as the dispersant.

#### 2.4.7 Thermogravimetric (TG)

The thermogravimetry (TG) and differential thermogravimetry (DTG) curves of pectin were obtained using a thermal analyzer (DHG‐60H, Shimadzu Co. Ltd., USA) according to the methods of Liang et al. (2022). Approximately 5 mg of each pectin sample was put in an alumina crucible and measured under the flowing high purity N_2_ at 50 mL/min. The sample was heated from 50°C to 800°C at a rate of 10°C/min.

### 2.5 Structural Properties of Pectin

#### 2.5.1 Fourier Transform Infrared (FT‐IR) Spectra

The FT‐IR spectra of pectin were acquired using a Nicolet iS5 Fourier‐transform infrared spectrometer (Thermo Scientific, USA). A 1.0 mg sample was thoroughly ground with KBr (100 mg) and compressed into a transparent pellet under 50 MPa pressure for 5 min. Spectral data were collected in the 4000–400 cm^−1^ range, with the degree of esterification (DE) calculated according to the methodology established by Liu et al. (2022).

#### 2.5.2 Scanning Electron Microscopy (SEM)

The surface morphology of pectin powder was examined using a JSM‐5600LV scanning electron microscope (SEM, JEOL, Tokyo, Japan) following the sample preparation protocol established by Yan et al. (2021). Briefly, pectin specimens were mounted on aluminum stubs using conductive carbon tape and sputter‐coated with a 15‐nm gold–palladium layer under high vacuum to enhance surface conductivity. Microstructural characterization was performed in secondary electron imaging mode at an accelerating voltage of 5.0 kV, with representative images captured at standard magnifications of 500× and 2000× under high‐vacuum conditions.

### 2.6 Functional Characteristics of Pectin

#### 2.6.1 Rheological Properties

The rheological characteristics of pectin were investigated following the methodology described by Yu et al. (2021) with minor adaptations. Apparent viscosity measurements of the pectin solution (30 g/L) were performed using an AR1500EX rotational rheometer (TA Instruments, USA) configured with a 40 mm parallel plate geometry (1.0 mm gap). Shear rate‐dependent viscosity was analyzed through controlled shear rate ramps from 0.1 to 100 s^−1^ at 25°C.

The viscoelastic properties were determined via oscillatory frequency sweep tests (0.1–10 Hz) under 5% strain amplitude at 25°C, maintaining measurements within the linear viscoelastic region (LVR). Prior to frequency sweeps, strain amplitude optimization was conducted through amplitude sweep tests at 1.0 Hz to establish the LVR threshold.

#### 2.6.2 Antioxidant Activity

The DPPH radical scavenging capacity of pectin was evaluated using a modified protocol from Chen et al. (2022). In brief, 2 mL of pectin solution (0–10.0 mg/mL) was combined with 2 mL of 0.2 mM DPPH ethanolic solution in test tubes. The reaction system was vortex‐mixed and subsequently incubated in darkness at 25°C for 30 min. Absorbance was measured at 517 nm using a UV–vis spectrophotometer, with absolute ethanol serving as the blank control. Ascorbic acid (0.1 mg/mL) was employed as the reference antioxidant in parallel experiments.

The DPPH scavenging activity was evaluated using Equation (5).

(5) $$\text{DPPH radical scavenging activity}\;\left(\%\right)=\left[1+\left(A_{1}-A_{2}\right)/A_{0}\right]\times 100$$

where *A*_0_ and *A*_1_ were the absorbances of DPPH solution and ethanol instead of DPPH solution, respectively, *A*_2_ was the absorbance of sample and DPPH solution.

The ABTS^+^_˙_ scavenging activity of pectin was evaluated following a modified protocol adapted from Liu et al. (2022). The ABTS^+^_˙_ radical solution was prepared by mixing 7 mM ABTS with 2.45 mM potassium persulfate, followed by overnight incubation at 25°C in the dark. The resulting solution was then diluted with ethanol to achieve an absorbance of 0.70 ± 0.02 at 734 nm. For analysis, 2.0 mL of pectin solutions (0–10 mg/mL) were mixed with 4.0 mL of the ABTS^+^_˙_ solution and maintained in dark conditions at 25°C for 10 min before measuring the absorbance at 734 nm. Ascorbic acid served as the positive control in parallel experiments. The ABTS^+^_˙_ scavenging activity was determined using Equation (6).

(6) $${\text{ABTS}}^{+}˙\text{scavenging activity}\;\left(\%\right)=\left[\left(A_{1}-A_{2}\right)/A_{1}\right]\times 100$$

where *A*_1_ and *A*_2_ were the absorbances of ABTS^+^_˙_ solution without pectin and ABTS^+^_˙_ solution with pectin, respectively.

#### 2.6.3 Heavy Metals Adsorption Capacity

The heavy metals (Cu^2+^, Zn^2+^, Mg^2+^, Pb^2+^, Cr^3+^, and Cd^2+^) were used to measure the adsorption capacity of pectin using the method of Shao et al. (2021) with minor modifications. Briefly, 50 mg pectin was reacted with 40 mL metal solutions (200 mg/mL) at 37°C for 2 h, and the mixtures were centrifuged at 3773 *g* for 20 min and filtered through a 0.45 μm filter membrane. Finally, the initial and final metal ion concentrations were measured by inductively coupled plasma mass spectrometry (ICP‐MS, Agilent 7800, Agilent Technologies, USA).

### 2.7 Statistical Analysis

The experimental data were obtained from triplicate determinations and expressed as mean ± SD. Statistical differences were analyzed using one‐way ANOVA with Duncan's test (*p* < 0.05) through IBM SPSS Statistics (version 23). Pearson correlation analysis was performed to establish relationships among pectin's structural characteristics, functional properties, and antioxidant activities. A gradient color matrix was employed to visualize correlation coefficients, with red hues (R = +1) representing strong positive correlations and blue hues (R = −1) indicating negative correlations, while color intensity reflected the magnitude of association.

## 3 Results and Discussion

### 3.1 Physicochemical Properties of MPP

#### 3.1.1 Yield and Degree of Esterification (DE) of MPP

**FIGURE 1:**
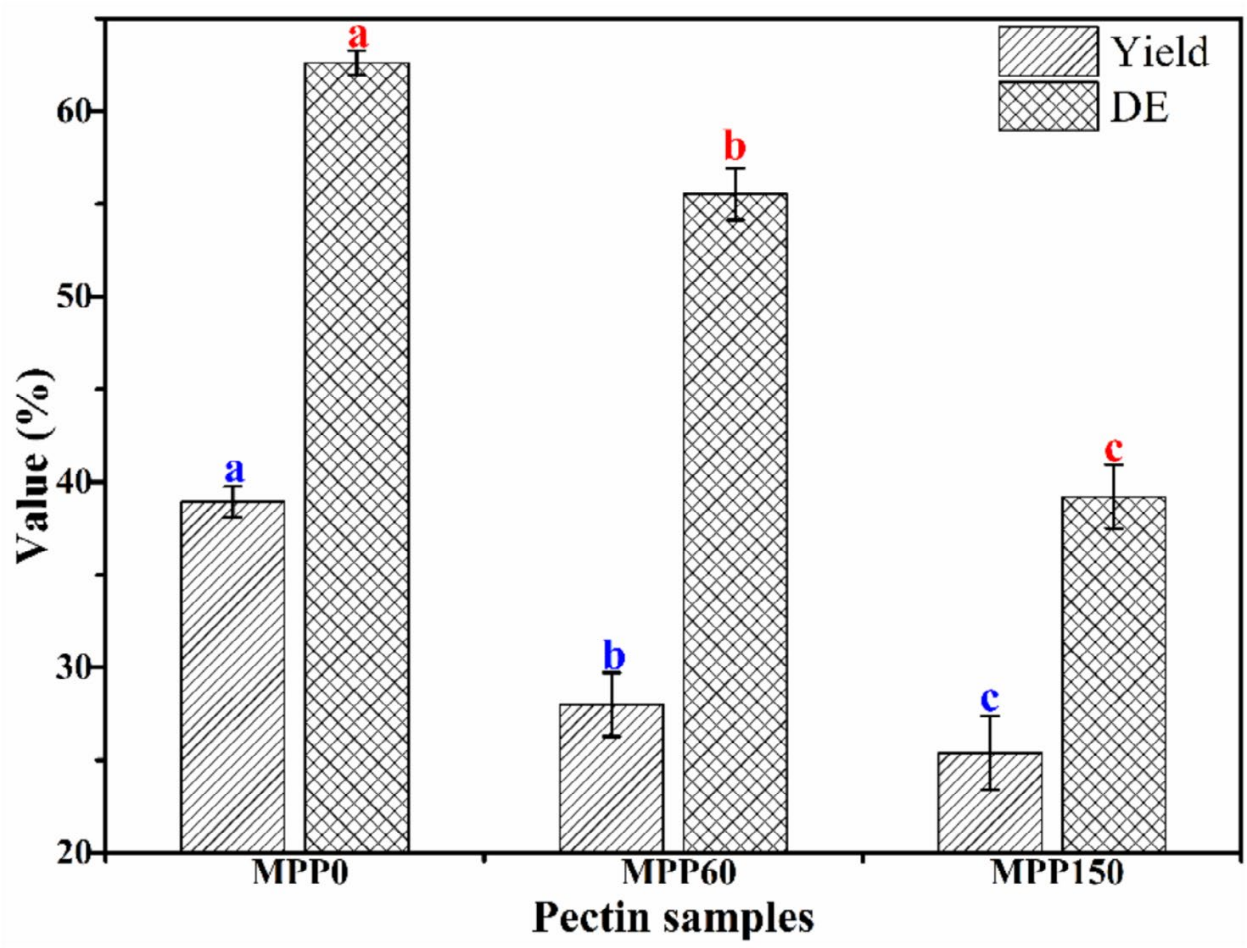
The yield and degree of esterification (DE) of Majiayou pomelo peel pectin (MPP) from Majiayou pomelo peel (MP) with different storage periods.

Figure 1 displays that the yield of pectin extracted from Majiayou pomelo peel (MP) was over 25% using the extraction method of citric acid, which was higher than that of passion fruit peel (22.63%) (Huo et al. 2023) and orange peel (22.8%) (Zioga et al. 2022). Moreover, the yield of MPP gradually decreased with the increase of pomelo storage periods. The yield of MPP_0_ could obtain the highest value (38.92%), and then decreased to 25.38% after 150 days storage of Majiayou pomelo because of the degradation of soluble pectin, which was consistent with the previous reports (Frempong et al. 2022; Chandel et al. 2022). Frempong et al. (2022) reported that pectin content from tarocco blood orange significantly decreased by 42.1% after 63 days storage. These results indicated that the yield of pectin from fruit peel was substantially affected by postharvest storage time and raw material.

The DE of pectin is a vital parameter influencing its quality and application (Zioga et al. 2022). Figure 1 showed that MPP_0_ (DE value of 62.61%) could be considered HMP (DE > 50%). The DE value of MPP exhibited a progressive decline with prolonged storage, indicating ongoing de‐esterification processes. This phenomenon primarily resulted from methyl group (–CH_3_) depletion coupled with enzymatic cleavage of inter‐pectate ester bonds mediated by endo‐polygalacturonase (endo‐PG) activity (Chandel et al. 2022; Jiang et al. 2020). The de‐esterification of MPP could lead to the loose and softening of the cell walls in MP. The DE value of MPP gradually decreased to 55.52% at 60 days storage and further decreased to the range of LMP (39.22% < 50%) after 150 days storage, indicating that MPP from MP within 60 days storage could be employed as high‐sugar and gel foods within, if over 60 days storage, it was usually used in low‐sugar foods.

#### 3.1.2 Molecular Weight, Zeta Potential, and Particle Distribution

**FIGURE 2:**
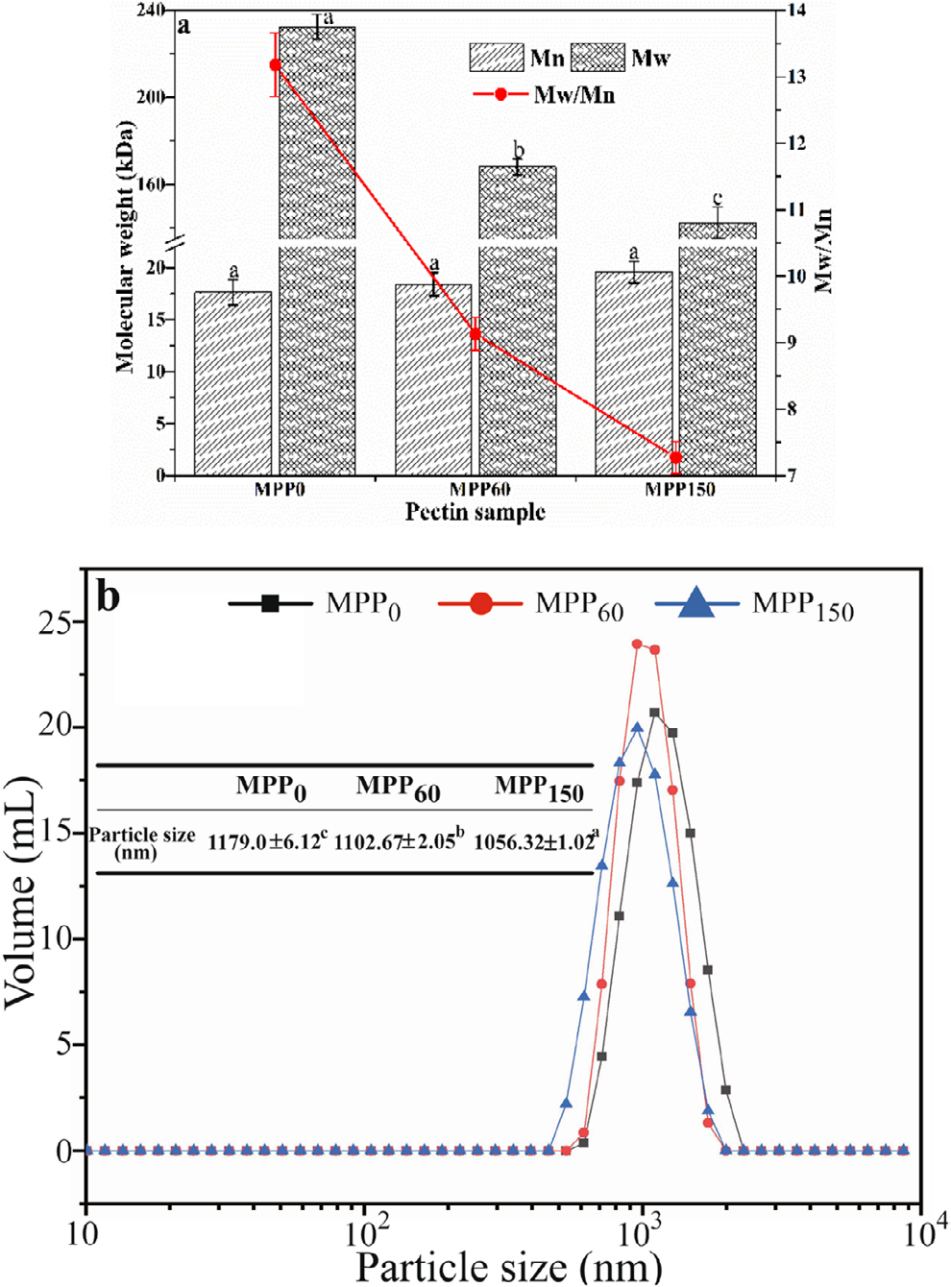
The molecular weight (Mw, a) and particle size (b) of Majiayou pomelo peel pectin (MPP) from Majiayou pomelo peel (MP) with different storage periods.

The Mw is closely correlated to the functional features and application of pectin (Huo et al. 2023), such as gel strength, viscosity, stabilizer, emulsifier, etc. The Mw distribution of MPP extracted from MP with different storage periods was also depicted in Figure 2a. The molecular weight (Mw) of MPP showed a progressive decrease with extension of pomelo storage periods, a phenomenon potentially attributed to β‐elimination‐mediated chain scission of the pectin backbone during prolonged storage (Liang et al. 2022). The Mw of MPP significantly declined from 232.47 to 168.08 kDa after 150 days storage (*p* < 0.05). The polydispersity index (PDI) is regularly used to represent the Mw distribution of polymers mixture. The smaller the PDI, the more concentrated the Mw distribution, and the lower the degree of mixing of the long and short chains (Jiang et al. 2020). Figure 2a presents that the PDI of MPP gradually declined with a reduction in the pomelo freshness, indicating that the Mw distribution of MPP has become narrower during storage. The MPP_0_ (PDI = 13.18) showed a long and intact molecular chain, leading to the larger Mw and PDI value. After 150 days of storage, MPP_150_ displayed a reduced polydispersity index (PDI) of 8.17, likely driven by β‐elimination‐induced chain scission. This degradation mechanism accelerated cleavage of both main and side chains, homogenizing molecular weight distribution as fragmented chains converged toward a uniform length profile.

Zeta potential of pectin is used to assess its stability and emulsion activity. The higher the absolute zeta potential indicated the more stable pectin solution (Liu et al. 2023). Table 1 presents the zeta potential of MPP from different freshness MP. The MPP was anionic polysaccharide with negative potential due to the COO– in pectin chains (FTIR spectra in Section 3.2), and the absolute zeta potential of MPP gradually increased with a decrease in pomelo freshness. The absolute zeta potential of MPP_0_ was the lowest value (26.41 mV) and then significantly increased to 34.10 mV after 150 days of storage (*p* < 0.05) probably due to an increase in negative charges from –COOH induced by de‐esterification during storage of Majiayou pomelo, suggesting that MPP from no fresh MP could present excellent stability and emulsifying capacity by electrostatic repulsion to prevent coalescence and flocculation phenomena (Zhang et al. 2023). Figure 2b shows that the particle size of MPP gradually decreased from 1179 to 1056 nm after 150 days of storage of Majiayou pomelo due to an increase in molecular electric charge repulsion caused by an increase in absolute Zeta potential, indicating the increase in pectin stability during storage.

**TABLE 1:** The structural characteristics of pectin and color difference of Majiayou pomelo peel pectin (MPP) from Majiayou pomelo peel (MP) with different storage periods.

Samples	MPP_0_	MPP_60_	MPP_150_
Homogalacturonan (HG, %)	71.98 ± 2.36^a^	68.67 ± 2.58^b^	64.81 ± 2.25^b^
Rhamnogalacturonan‐I (RG‐I, %)	21.33 ± 1.02^b^	25.05 ± 0.95^a^	28.36 ± 1.36^a^
Rhamnose/Galacturonic acid (Rha/GalA)	0.04 ± 0.003^a^	0.05 ± 0.002^a^	0.06 ± 0.001^a^
(Galactose+Arabinose)/Rhamnose ((Gal +Ara)/Rha)	4.64 ± 0.18^b^	4.92 ± 0.36^b^	5.18 ± 0.65^a^
Zeta potential (mV)	−26.41 ± 0.69^c^	−28.67 ± 1.42^b^	−34.10 ± 1.13^a^
Color			
Lightness (L*)	76.87 ± 0.35^b^	77.37 ± 0.13^a^	77.56 ± 0.10^a^
Redness (a*)	−1.12 ± 0.02^a^	−1.09 ± 0.01^b^	−1.07 ± 0.01^c^
Yellowness (b*)	−2.77 ± 0.67^a^	−3.50 ± 0.17^ab^	−3.64 ± 0.07^b^
Δ*E*	0^c^	0.89 ± 0.15^b^	1.12 ± 0.01^a^

*Note:* Different lowercase letters in table indicate significant differences (*p* < 0.05).

#### 3.1.3 Monosaccharide Composition

**FIGURE 3:**
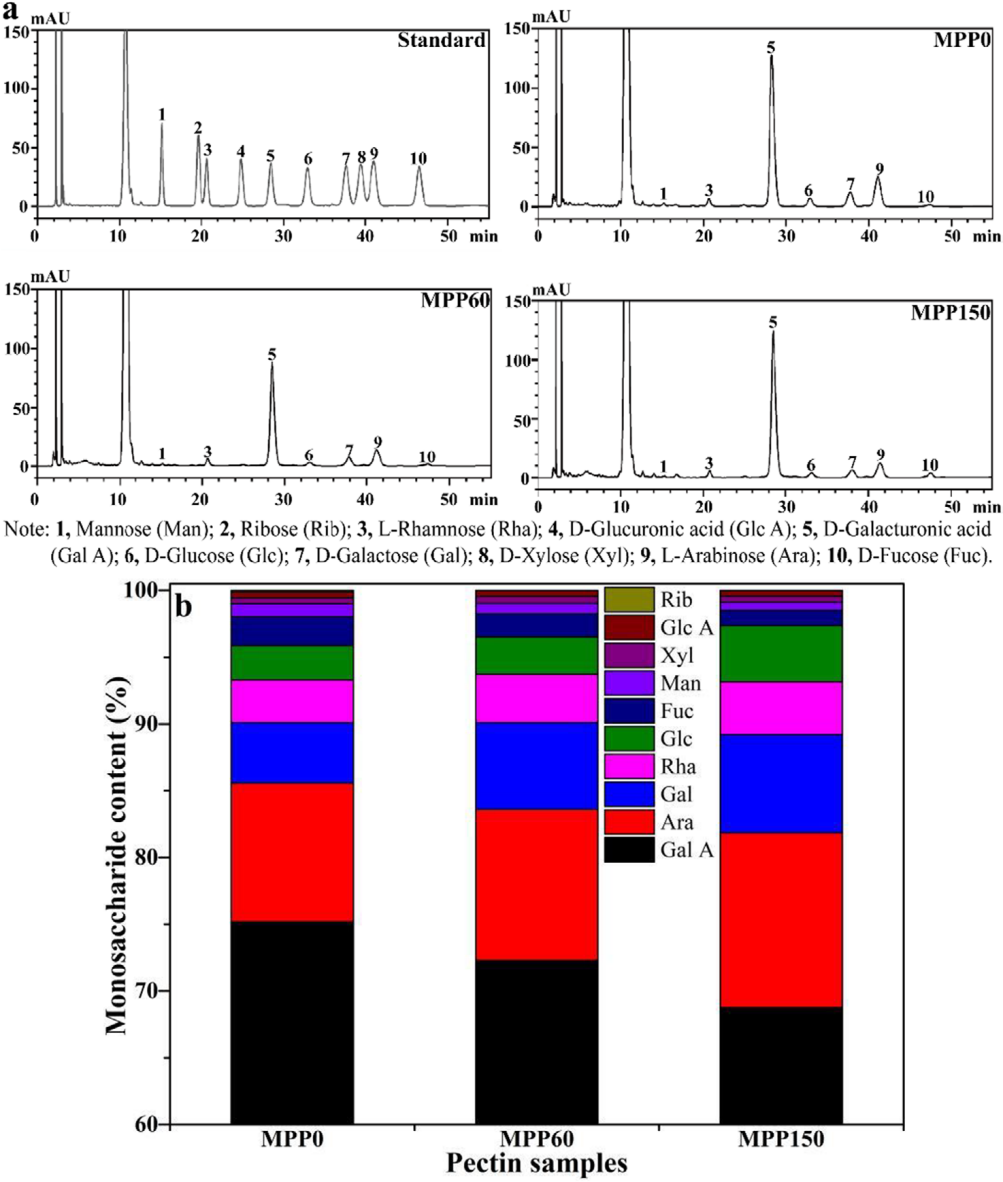
The HPLC diagrams (a) and monosaccharide content (b) of Majiayou pomelo peel pectin (MPP) from Majiayou pomelo peel (MP) with different storage periods.

The GalA linear chain serves as the backbone of pectin, interspersed with sporadic sugar units or short‐chain branches. Figure 3 illustrates the HPLC profiles and monosaccharide composition of MPP derived from MP with different storage periods. As shown in Figure 3a, MPP primarily consists of galacturonic acid (GalA), rhamnose (Rha), mannose (Man), galactose (Gal), glucose (Glc), arabinose (Ara), and fucose (Fuc). While the monosaccharide profiles of MPP from MP with different storage periods showed no significant variations, their relative abundances differed markedly (Figure 3b). GalA dominated the monosaccharide composition, accounting for 75.19 mol% in MPP_0_, followed by Ara (10.38%), Gal (4.53%), Rha (3.21%), and Glc (2.56%). This hierarchical order (GalA>Ara>Gal>Rha>Glc) remained consistent across all samples, highlighting GalA's structural predominance in pectin. GalA constitutes a linear backbone of pectin, Gal and Ara comprise of the side chains of Rhamnogalacturonan‐I (RG‐I), whereas Fuc and Man are probably side chains of Rhamnogalacturonan‐II (RG‐II) (Huo et al. 2023). The GalA content of MPP gradually declined with the reduce of pomelo freshness, decreasing to 68.76 mol% after 150 days of storage possibly due to fracture of long chain by splitting HG backbone caused by endogenous enzymes during the storage of Majiayou pomelo, whereas the Gal and Ara content gradually increased during the storage of Majiayou pomelo, indicating that the breakage of RG‐I of MPP to enhance the ratio of side chain in single molecule chain during storage of Majiayou pomelo (Fihry et al. 2022). As shown in Table 1, during 150 days of storage, MPP underwent dynamic structural reorganization with opposing trends in its two main domains. Homogalacturonan (HG, linear backbone) decreased from 71.98% to 64.81% (−9.96%, *p* < 0.05), whereas rhamnogalacturonan‐I (RG‐I, branched regions) increased from 21.33% to 28.36% (+32.96%, *p* < 0.01). This HG/RG‐I ratio inversion (3.37 → 2.29) likely resulted from β‐elimination, residual polygalacturonase activity, and transglycosylation‐driven reassembly, which may critically impair pectin's gelling capacity and hydration properties (Fihry et al. 2022; Wang et al. 2016). The Rha/GalA and (Gal+Ara)/Rha ratios in MPP increased to 0.06 and 5.18, respectively, as the storage period of pomelo increased, suggesting that the prolonged storage period may enhance the emulsifying activity and emulsion stability of MPP through endogenous enzyme‐mediated depolymerization of its main chains (Jiang et al. 2020). The presence of glucose (Glc) and xylose (Xyl) in MPP might be attributed to the attachment of cellulose and hemicellulose to pectin (Wang et al. 2016).

The color stability of pectin solutions significantly influences gel and food product appearance, a crucial factor in consumer acceptance. As shown in Table 1, 1% MPP solutions maintained consistent color parameters throughout 150‐day storage: lightness (L*, 76.87–77.57), redness (a*, −1.12 to −1.02), and yellowness (b*, −3.64 to −2.77) showed no significant variations. However, the color difference (Δ*E*) progressively increased from 0 to 2.12 with the increase of the pomelo storage period, revealing measurable surface color changes. This phenomenon likely stems from oxidative degradation of phenolic compounds and carotenoids encapsulated within MPP during storage, as previously documented in citrus systems (Wang et al. 2016). The increase of gradual Δ*E* suggests that while basic color coordinates remain stable, subtle chemical alterations accumulate over time, potentially affecting product quality perception.

#### 3.1.4 Thermal Stability

**FIGURE 4:**
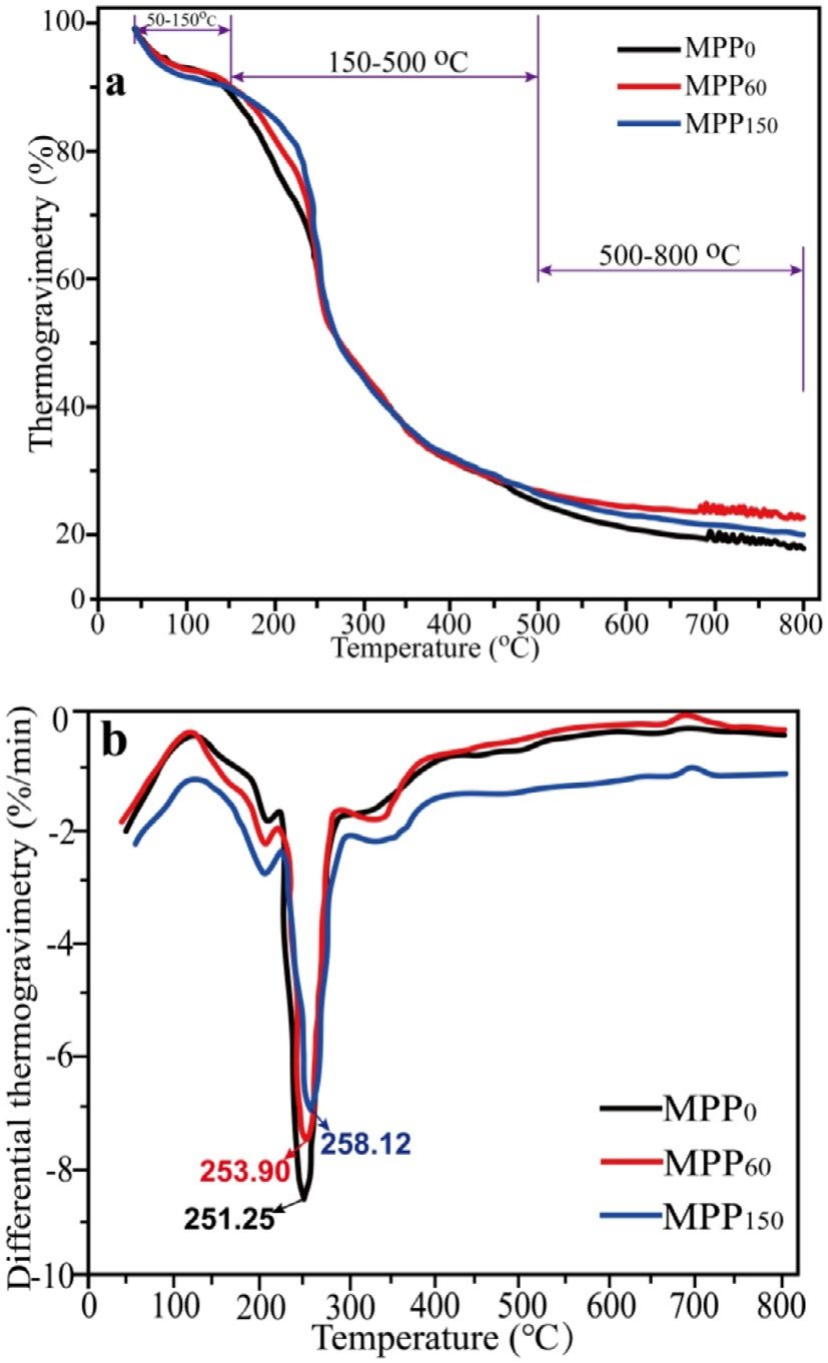
Thermogravimetry (TG, a) and differential thermogravimetry (DTG, b) curves of Majiayou pomelo peel pectin (MPP) from Majiayou pomelo peel (MP) with different storage periods.

The thermogravimetry (TG) analysis is frequently used to study the structure changes and thermal stability, which directly affect its application fields (Liang et al. 2022). The TG and DTG curves of pectin are depicted in Figure 4. TG curves mainly showed the relationship between mass and temperature. Figure 4a shows that all pectin has similar variation and can be divided into three decomposition stages: 50°C–150°C, 150°C–500°C, and 500°C–800°C. The mass loss (~10%) between 50°C and 150°C was mainly attributed to the evaporation of free water adsorbed in pectin by hydrogen bonds. The continuous weight loss (~65%) at 150°C–500°C was due to the decomposition of pectin, including the formation of various gases and solid substances with the chain breaking and depolymerization of structure (Liang et al. 2022). When the temperature was over 500°C, the mass loss became slower. The minor weight loss (about 5%) was induced by the secondary thermal decomposition of solid substances (including alphatic and ketone group grafted polyaromatic hydrocarbon) at 500°C–800°C (Grassino et al. 2016). Up to 800°C, the residual mass of MPP_60_ and MPP_150_ was 22.79% and 20.09%, respectively. The weight loss of MPP_0_ (81.99%) from fresh pomelo peel was greater than that of MPP_60_ (77.21%) and MPP_150_ (79.91%) from postharvest storage.

The differential thermogravimetry (DTG) curve is the first derivative of the TG curve with respect to time, representing the relationship between the rate of mass loss and time. Generally, low TP and high absolute Vmax in the DTG curve could indicate a low thermal stability of MPP. Figure 4b presents the basically similar DTG curve of all pectin groups in shape. The MPP from fresh MP (MPP_0_) had the lowest temperature peak (TP) and maximum reaction velocity (*V*_max_) (−8.62%/min). The TP value of MPP gradually increased from 251.25°C to 258.12°C after 150 days of storage, and its absolute *V*_max_ value also gradually decreased with the increase of postharvest pomelo storage time, indicating that the thermal stability of MPP gradually enhanced with the reduction of pomelo freshness, which may be due to the conformational transition of molecular structure and the fracture of the pectin main chain (Liang et al. 2022).

### 3.2 FTIR And SEM Analysis

**FIGURE 5:**
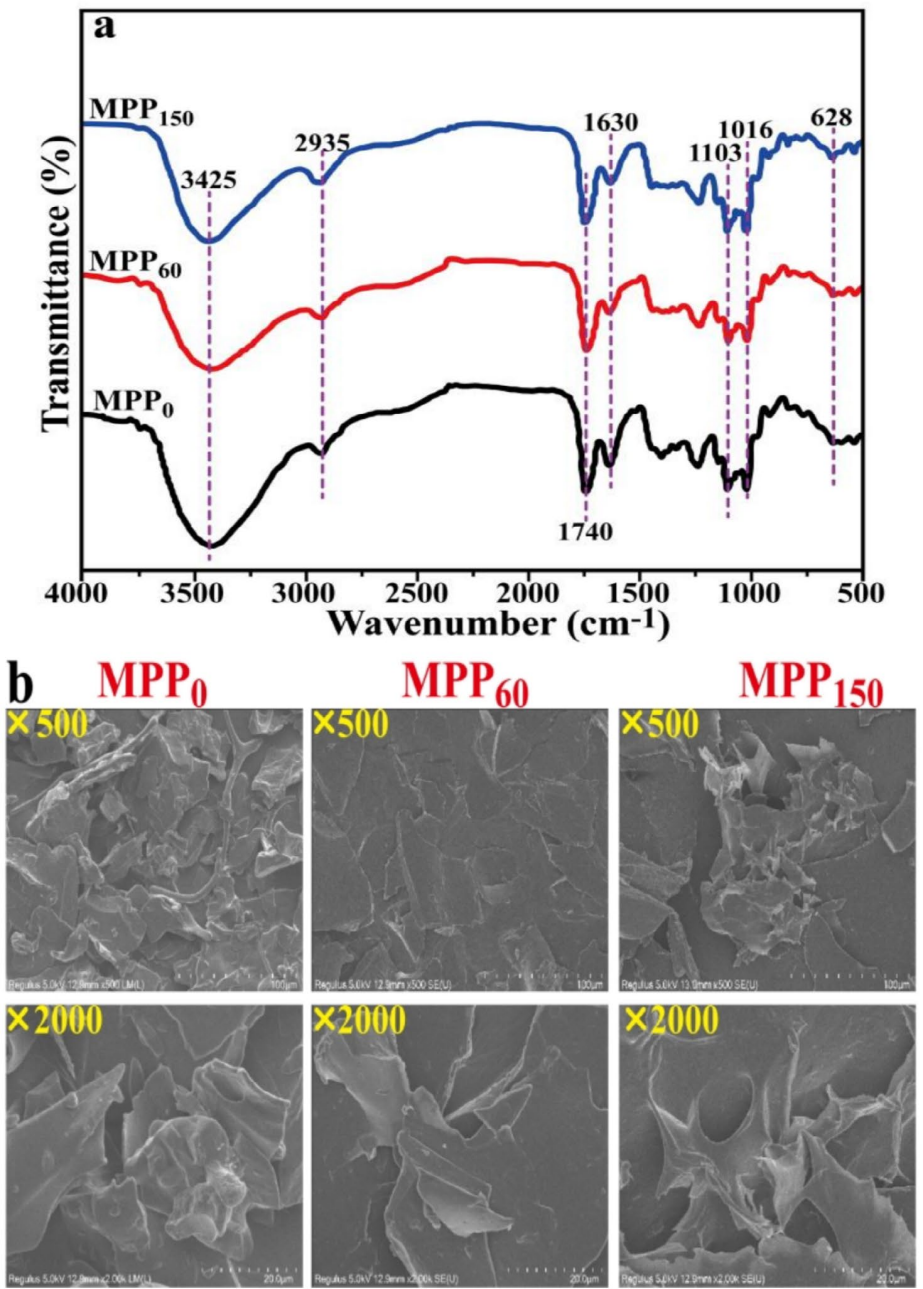
The Fourier transform infrared (FTIR, a) spectra and scanning electron microscopy (SEM, b) images of Majiayou pomelo peel pectin (MPP) from Majiayou pomelo peel (MP) with different storage periods.

The fourier transform infrared (FTIR) spectra of MPP from different freshness MP are shown in Figure 5a. The FTIR spectra of MPP had similar characteristic absorption peaks, presenting that the primary structures of pectin were no obvious change during the storage of pomelo. The absorption peaks area at 3425 cm^−1^, attaching to stretching vibrations of –OH groups in MPP (Jiang et al. 2020), which significantly decreased with the extension of postharvest pomelo storage. The absorption peaks at 2935 cm^−1^ attributing to the asymmetrical and symmetrical –C–H of the aliphatic carbon galacturonic ring have no obvious difference during 150 days of storage of pomelo (Grassino et al. 2016). The characteristic signals at 1740 and 1630 cm^−1^ were due to the –C=O stretching vibration of –O–C=O groups and –C=O asymmetrical stretching vibration of –COOH groups, respectively. The absorption peak in the carboxyl response area of MPP gradually weakened with the decrease of pomelo freshness, which directly affected the functional properties of pectin, including gelation activity, emulsifying ability, and metal binding capacity (Liu et al. 2022). In general, the bands from 600 to 1300 cm^−1^ was regularly considered “fingerprint region” of pectin, in which different types of pectin could be recognized. The absorption peaks area of MPP at 1103 and 1016 cm^−1^ had no significant distinction during 150 days storage of pomelo, corresponding to the stretching vibrations of –C–OH side groups and –C–O–C glycosidic bonds vibration, respectively. The absorption peak at 628 cm^−1^ was gradually strengthened with the decrease of pomelo freshness, assigning to low‐frequency vibrations of the pyranose ring and the deformation vibrations of the C–C ring skeletal in pectin (Liu et al. 2022; Hu et al. 2022).

Figure 5b reveals the characteristic lamellar microstructure of pectin through scanning electron microscopy (SEM) analysis. All samples exhibited wrinkled, irregular flake structures with surface cracks attributable to ice sublimation during freeze‐drying (Liu et al. 2022). Notably, MPP from pomelo with an increase in the storage period showed progressive thinning and fragmentation of surface structures. Comparative analysis demonstrated that while MPP_0_ possessed loose, thick lamellae, both MPP_60_ and MPP_150_ developed denser, smoother layered architectures with enhanced gel strength. This structural improvement correlates with increased surface area and optimized pore configuration, where the tightly interwoven network provides superior resistance to deformation.

### 3.3 Functional Characteristics of MPP

#### 3.3.1 Rheological Properties

**FIGURE 6:**
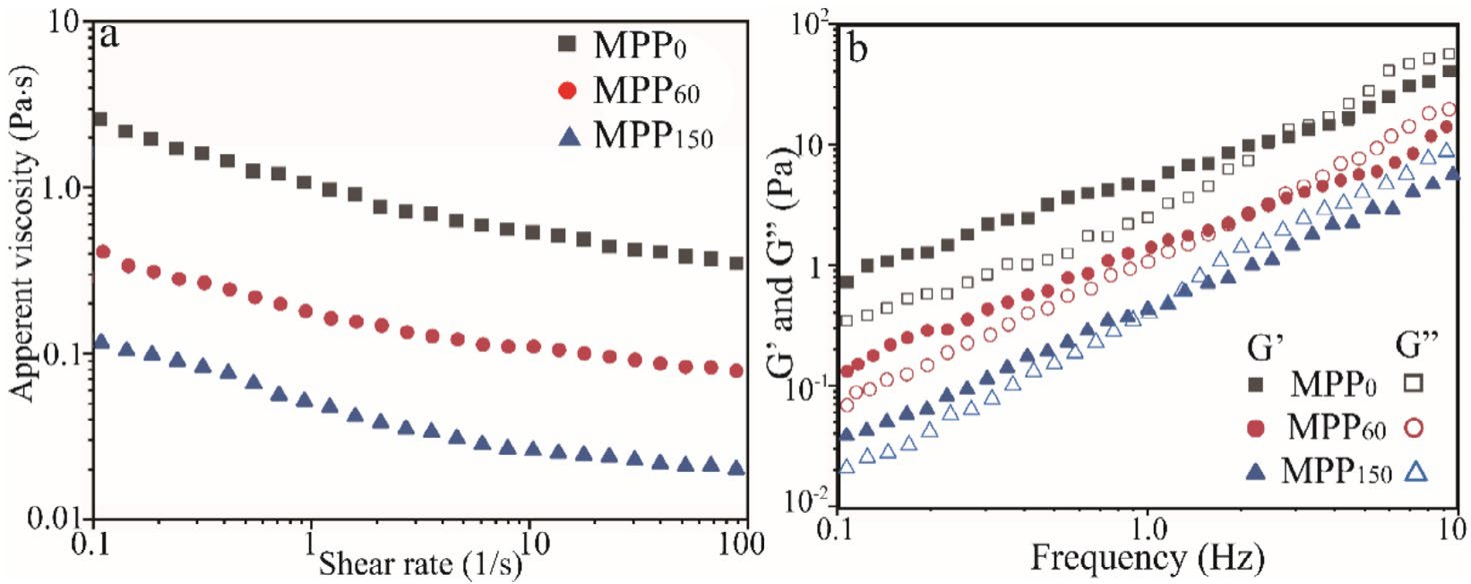
Rheological properties of Majiayou pomelo peel pectin (MPP) from Majiayou pomelo peel (MP) with different storage periods at a concentration of 30 mg/mL. Apparent viscosity (a) and Frequency scanning (b) of MPP.

Rheology is widely used to depict the flow properties of pectin in its technological properties and food applications. Figure 6a shows the relationship between shear rate and apparent viscosity of MPP from MP with different storage periods at 30 mg/mL and 25°C. The apparent viscosity of MPP gradually decreased with increasing shear rate from 0.1 to 100 s^−1^, which exhibited shear‐thinning behavior and non‐Newtonian pseudoplastic fluids due to the reduction of flow resistance and the entanglement between adjacent chains by increasing shear rate and promoting a more orderly directional arrangement of long‐chain molecules (Wang et al. 2019; Cui et al. 2020). Moreover, the apparent viscosity of MPP gradually declined with the reduction of pomelo freshness because of the decrease of DE value and GalA content during storage. MPP_0_ exhibited the highest apparent viscosity at the same shear rate because of its highest DE value and GalA content, indicating that the freshness of raw materials has a substantial influence on the viscosity of pectin. Previous reports showed pectin with high DE and intertwine could produce high hydrophobic interaction capacity with enhancement of viscosity (Yan et al. 2021; Bai et al. 2020).

Figure 6b presents that the variations in the elastic modulus (*G*′) and viscous modulus (*G*″) as a function of angular frequency scanning for MPP solutions. Both *G*′ and *G*″ of MPP solutions closely depended upon angular frequency (0.1–10 Hz). As the reduce of pomelo freshness, the *G*′ and *G*″ of MPP gradually declined due to the decrease of DE value MPP, implying the decrease of the viscoelasticity of MPP. The *G*′ of MPP solution was greater than its *G*″ at lower angular frequencies, presenting a weak colloid state based on elastic property (*G*′ > *G*″) (Zhang et al. 2023). The *G*″ of MPP solution was greater than its *G*′ at high angular frequencies, indicating a predominance of viscosity. In general, the higher of crossover point, the larger the dedication of elasticity (Liang et al. 2022). The crossover points of MPP gradually decreased with the increase of postharvest pomelo storage time, implying that the deformation resistance of MPP gradually weakened in the time‐dependent during postharvest pomelo storage possibly due to the breakage of long molecular chain and demethylation reactions (Li et al. 2022).

#### 3.3.2 Antioxidant Activity and Metals Adsorption Capacity

**FIGURE 7:**
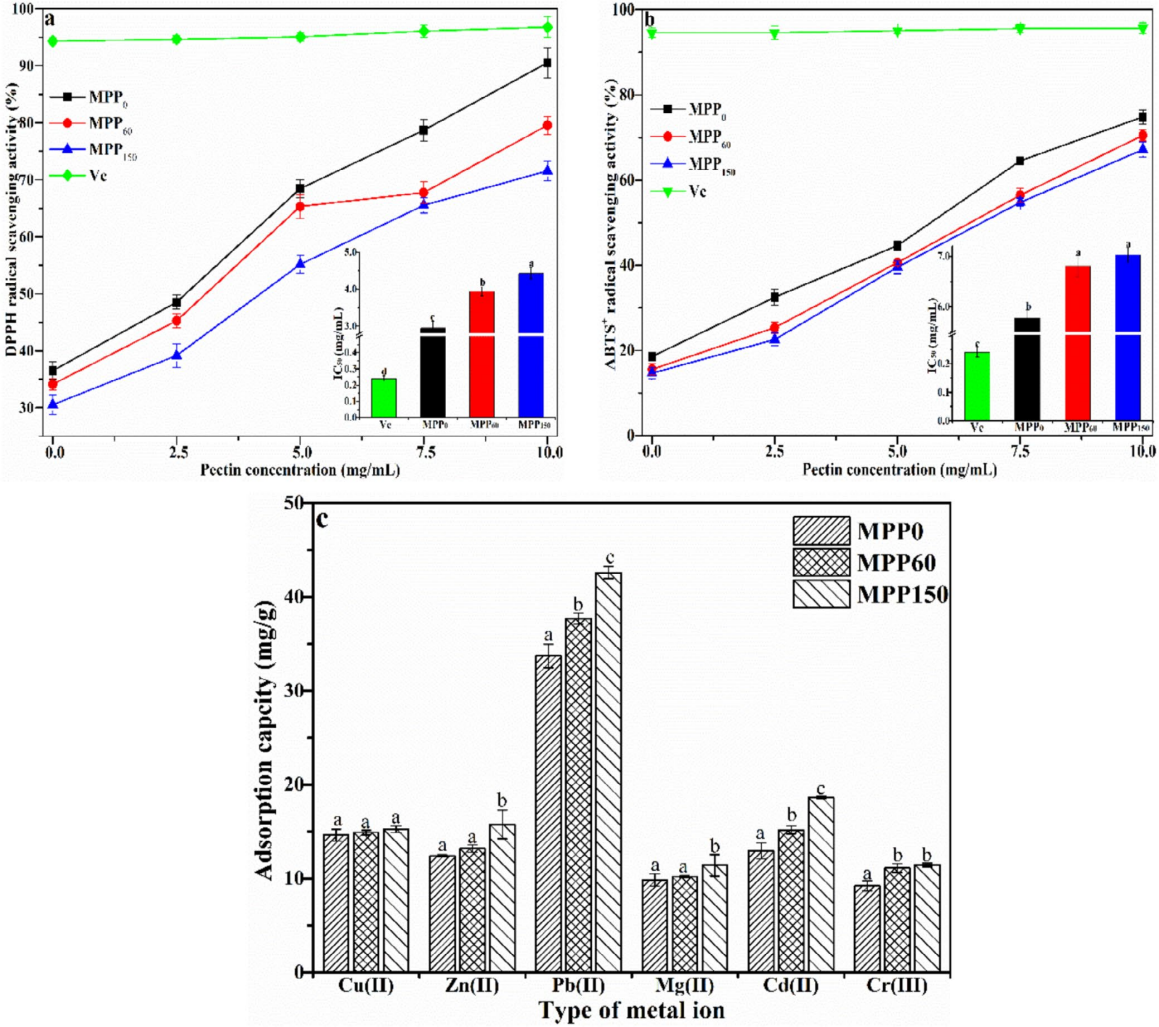
Antioxidant activity. (a) DPPH radical scavenging activity, (b) ABTS^+^˙ scavenging activity and metal adsorption capacity (c) of Majiayou pomelo peel pectin (MPP) from Majiayou pomelo peel (MP) with different storage periods.

The antioxidant activity (AOA) of MPP was assessed by DPPH and ABTS^+^˙ scavenging activities in vitro chemical assays. Figure 7a shows the DPPH radical scavenging activities of MPP from MP with different storage periods at 0–10.0 mg/mL. All MPP presented a concentration‐dependent activity to scavenge the DPPH radical. The DPPH scavenging capacity of MPP_0_, MPP_60_, and MPP_150_ reached 89.83%, 79.41%, and 71.17% at 10.0 mg/mL, respectively, indicating that the –OH group in MPP can devote hydrogen to scavenge the DPPH radical. Moreover, the DPPH scavenging activity of MPP_0_ was stronger than that of MPP_60_ and MPP_150_, but still slightly lower than that of Vc. The 50% inhibiting concentration (IC_50_) of MPP_0_ (2.95 mg/mL) was lower than that of MPP_60_ (3.87 mg/mL) and MPP_150_ (4.43 mg/mL), suggesting that MPP_0_ has a better capacity to remove DPPH radicals, probably because it contains a higher amount of hydroxyl and free carboxylic groups in MPP from fresh MP (Gao et al. 2023; Song et al. 2019).

ABTS^+^˙ scavenging activity is often used to characterize the antioxidant ability by transforming hydrogen atoms into nonfree radical species. As seen in Figure 7b, the scavenging capacity of MPP on ABTS^+^˙ was similar to that of the DPPH radical. The ABTS^+^˙ scavenging capacity of MPP_0_ gradually increased from 18.65% to 74.41% as the content increased from 1.0 to 10.0 mg/mL. Moreover, the IC_50_ of MPP_0_ (5.91 mg/mL) was significantly lower than that of MPP_60_ (6.95 mg/mL) and MPP_150_ (6.92 mg/mL), indicating that MPP_0_ exhibited a higher ABTS^+^˙ scavenging ability than that of MPP_60_ and MPP_150_, which may be due to the decrease of the DE value and Mw in MPP during storage (Yu et al. 2021; Song et al. 2019). These findings demonstrated the promising potential of fresh MP (MPP_0_) as a natural antioxidant for functional food and pharmaceutical applications.

Previous studies reported that pectin exhibited an optimistic affinity for metal ions by physically binding with divalent cations to form an insoluble “eggbox” hydrogel structure (Wang et al. 2019; Zhang et al. 2020). Figure 7c presents the adsorption ability of MPP from MP with different storage periods on a variety of metal ions. The adsorption capacity of MPP on metal ions presented the following order: Pb(II) > Cd(II) > Cu(II) > Zn(II) > Mg(II) > Cr(III), which could be related to the electronegativity and maximum mass charge ratio of the metal ions. The maximum adsorption ability of MPP_0_ on Pb(II) could reach 33.72 mg/g through the hydroxyl groups and carboxylic groups in galacturonic acid. Moreover, the adsorption ability of MPP gradually enhanced with the reduction of pomelo freshness due to the increase of absolute Zeta potential (in Section 3.1.2). The adsorption capacity of MPP_150_ on Pb(II) increased to 42.6 mg/g after 150 days of storage of pomelo, which could be due to the decrease of DE and particle size of MPP during the storage of Majiayou pomelo (Ibarra‐Rodríguez et al. 2017).

### 3.4 Correlation Analysis

**FIGURE 8:**
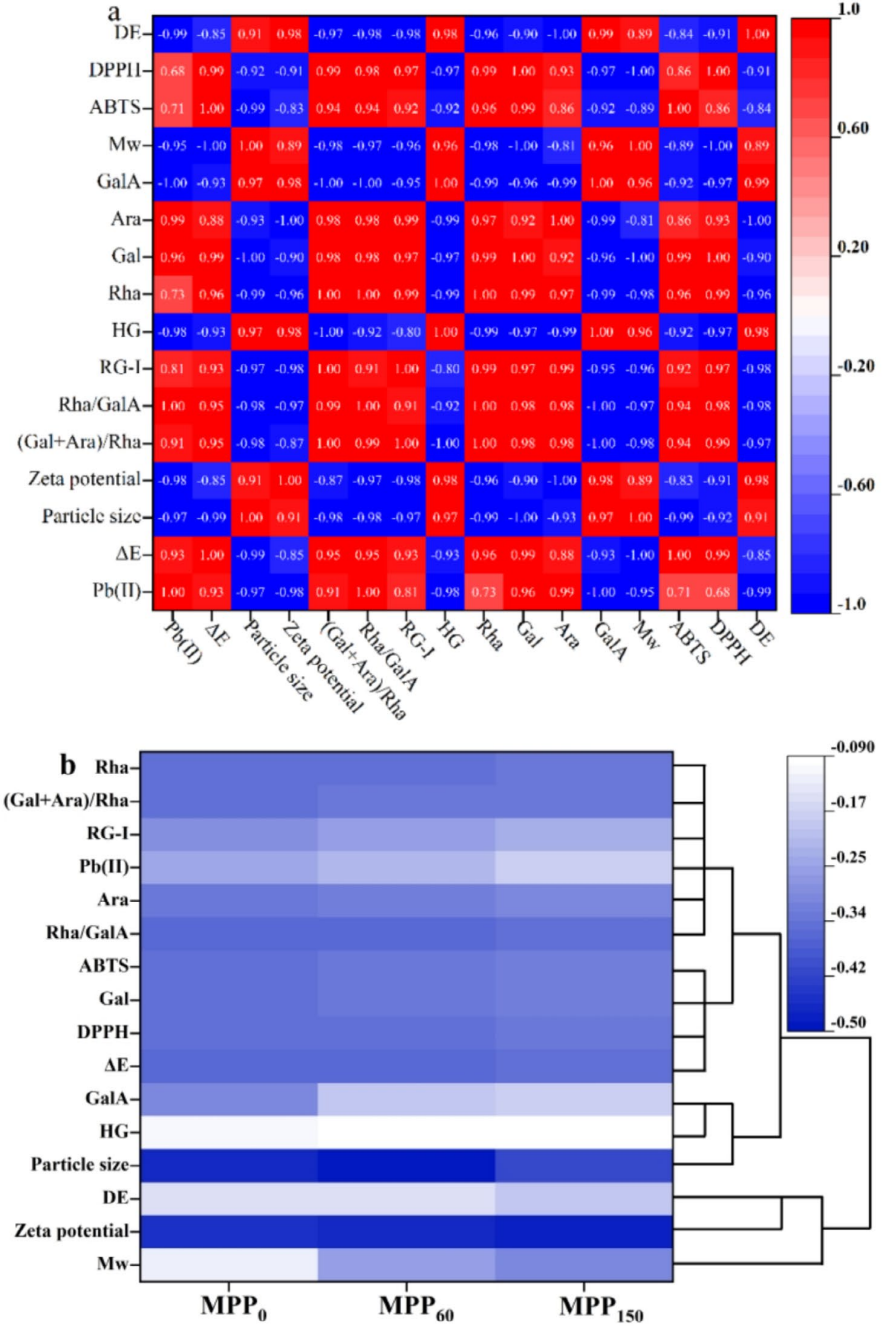
Analysis of Pearson's correlation (a) and clustering heat map (b) between structure and functional properties of Majiayou pomelo peel pectin (MPP) from Majiayou pomelo peel (MP) with different storage periods. From red to blue indicates the correlation from large to small, red indicates the positive correlation, and blue indicates the negative correlation.

As shown in Figure 8a, the antioxidant capacity (IC50) of MPP showed negative correlations with zeta potential, HG content, GalA, Mw, and DE, confirming that intact pectin main chains enhance antioxidant activity—a phenomenon consistent with citrus peel pectin studies (Liu et al. 2022). Enhanced antioxidant performance was specifically associated with higher (Gal + Ara)/Rha ratios, Gal, Ara contents, and RG‐I proportions, suggesting low‐branching RG‐I domains favor antioxidant efficacy. The metal adsorption capacity exhibited strong positive correlations with Rha/GalA (0.98) and neutral sugars (Gal:0.96, Ara:0.99, Glc:0.72), but inverse relationships with particle size (−0.97), DE (−0.98), GalA (−1.0), Mw (−0.95), and zeta potential (−0.98). This pattern highlights two key determinants: (1) carboxyl group density modulated by esterification degree and (2) improved specific surface area via reduced particle size (Wang et al. 2019). Notably, the complete inverse correlation with GalA (−1.0) underscores neutral sugar side chains' critical role in metal chelation.

In Figure 8b, MPP_0_ had high DE, GalA, Mw, DPPH, ABTS^+^˙ due to its relatively integrity of chemical structure. With the reducing of pomelo freshness, the gradual fracture of main chain in structure of MPP contributed to the decrease of its DE, antioxidant activity (AOA), and zeta potential, increase of adsorption capability. Therefore, MPP_150_ showed the highest values of metal adsorption and lowest antioxidant capacity, DE, GalA, Mw, (Gal+Ara)/Rha after 150 days of storage. To sum up, the correlation analysis suggested that the physiochemical, structural and functional properties of MPP were highly negative correlation with its freshness of raw materials.

## 4 Conclusion

This study systematically analyzed the impact of pomelo storage periods on the physicochemical and structural characteristics of Majiayou pomelo peel pectin (MPP). Results demonstrated progressive transformation of MPP from high‐methoxyl pectin (HMP) to low‐methoxyl pectin (LMP) as the storage period of pomelo increased. Concomitant reductions in yield, molecular weight, thermal stability, and antioxidant capacity (DPPH/ABTS radical scavenging capacity) were attributed to main chain fragmentation, evidenced by reduced galacturonic acid (GalA) content and degree of esterification. Notably, the RG‐I domain ratio (GalA/Rha) increased significantly, suggesting preferential preservation of rhamnogalacturonan structures during storage. The color difference, particle size, and attenuated rheological properties correlated with enhanced zeta potential and reduced intermolecular aggregation. Multivariate analysis revealed strong negative correlations between pomelo storage period and MPP's functional parameters. Key determinants for bioactivity included high DE, elevated GalA content, and limited RG‐I branching, which collectively enhanced antioxidant capacity and metal chelation efficiency. These findings highlight the critical role of raw material storage in modulating pectin functionality, advocating for future investigations into the underexplored biological activities of MPP, particularly its antimicrobial, hypolipidemic, and antidiabetic potentials.

## Author Contributions

**Jian Zhou:** writing – review and editing, supervision, investigation. **Shuoru Feng:** conceptualization, investigation, formal analysis. **Yue Zhou:** conceptualization, investigation, formal analysis. **Zhenghua Huang:** data curation, resources, software, writing – original draft. **Bin Li:** validation, visualization, writing – review and editing. **Mengting Pi:** validation, visualization, writing – review and editing. **Siyi Wei:** data curation, resources, software, writing – original draft. **Leipeng Cao:** project administration, supervision, methodology, writing – review and editing.

## Ethics Statement

The authors have nothing to report.

## Conflicts of Interest

The authors declare no conflicts of interest.

## Data Availability

All necessary data are provided in the article. However, the author agrees to share raw data upon request.
